# Effect of Hearing Aids on Auditory Function in Infants with Perinatal Brain Injury and Severe Hearing Loss

**DOI:** 10.1371/journal.pone.0041002

**Published:** 2012-07-13

**Authors:** Alma Janeth Moreno-Aguirre, Efraín Santiago-Rodríguez, Thalía Harmony, Antonio Fernández-Bouzas

**Affiliations:** Unidad de Investigación en Neurodesarrollo “Dr. Augusto Fernández Guardiola”, Instituto de Neurobiología, Universidad Nacional Autónoma de México (UNAM), Campus Juriquilla, Querétaro, México; Hôpital Robert Debré, France

## Abstract

**Background:**

Approximately 2–4% of newborns with perinatal risk factors present with hearing loss. Our aim was to analyze the effect of hearing aid use on auditory function evaluated based on otoacoustic emissions (OAEs), auditory brain responses (ABRs) and auditory steady state responses (ASSRs) in infants with perinatal brain injury and profound hearing loss.

**Methodology/Principal Findings:**

A prospective, longitudinal study of auditory function in infants with profound hearing loss. Right side hearing before and after hearing aid use was compared with left side hearing (not stimulated and used as control). All infants were subjected to OAE, ABR and ASSR evaluations before and after hearing aid use. The average ABR threshold decreased from 90.0 to 80.0 dB (p = 0.003) after six months of hearing aid use. In the left ear, which was used as a control, the ABR threshold decreased from 94.6 to 87.6 dB, which was not significant (p>0.05). In addition, the ASSR threshold in the 4000-Hz frequency decreased from 89 dB to 72 dB (p = 0.013) after six months of right ear hearing aid use; the other frequencies in the right ear and all frequencies in the left ear did not show significant differences in any of the measured parameters (p>0.05). OAEs were absent in the baseline test and showed no changes after hearing aid use in the right ear (p>0.05).

**Conclusions/Significance:**

This study provides evidence that early hearing aid use decreases the hearing threshold in ABR and ASSR assessments with no functional modifications in the auditory receptor, as evaluated by OAEs.

## Introduction

Perinatal brain injury (PBI) consists of a large group of conditions that produce mild to severe impairments in motor, visual, auditory and cognitive functions [1], [2]. Hearing loss affects 1 to 3 per 1000 normal newborns and 2 to 4 per 100 of infants who require neonatal intensive care [3], [4]. Early hearing loss detection can only be achieved with sensitive diagnostic techniques. Ideally, hearing loss screening should be performed before 3 months of age, and an appropriate intervention should be implemented no later than 6 months of age [5].

Currently, the most useful techniques for early hearing loss detection are auditory brainstem responses (ABRs) and otoacoustic emissions (OAEs); however, new techniques such as auditory steady state responses (ASSRs) have been developed. These techniques show high sensitivity for detecting hearing loss in healthy and PBI infants [6]–[10].

The three techniques mentioned above measure different aspects of auditory function. OAEs evaluate the activity of the cochlear outer hair cells (OHCs) [6]. ABRs assess the conduction properties of pathways from the auditory nerve to the inferior colliculus and can determine auditory threshold. On the other hand, it has been demonstrated that ABRs can only detect frequencies in a narrow range, perhaps between 2000–4000 Hz [11]–[13].

More recently, auditory steady-state responses (ASSR) have been reported as a reliable and objective technique for evaluating hearing thresholds, with the advantage that several frequency-specific thresholds can be assessed simultaneously, and one frequency-specific audiometry can be obtained [9], [14]–[17].

One treatment for hearing loss is the use of hearing aids (HA), and cochlear implants are employed in selected cases. However, severe and moderate hearing loss may not be identified until the second year of life, and mild hearing loss is usually not detected until the child begins school [18]. After the first year of life, the sensible epochs for language acquisition have elapsed. As a result, the prescription of auditory aids is most useful during the first year of life [19].

Therefore, our aim was to analyze the effects of HA use on auditory function as evaluated by modifications in OAEs, ABRs and ASSRs in a group of infants with PBI and severe, profound hearing loss.

## Methods

Infants with PBI attending a specialized neurodevelopment research unit were included in this study. PBI was suspected when an injury that occurred between the 28^th^ week of gestation and 28 days after birth gave rise to abnormal neurological examination and abnormal magnetic resonance image (MRI) findings. All infants underwent a neurological examination in addition to the following evaluations: otoscopy, tympanometry, acoustic stapedial ipsilateral reflex (ASIR), OAEs, ABR, ASSRs and MRI. A total of 13 infants with severe or profound bilateral sensorineural hearing loss were included in this study. The parents of all infants provided written informed consent, and the study was approved by the institutional review board of the Institute of Neurobiology, National Autonomous University of México.

The tympanometry test was carried out using MT10 equipment (Interacocustics, Denmark), with a base frequency of 1000 Hz (high frequency tympanometry), maximum pressure of 200 daPa, minimum pressure of −200 daPa, maximum compliance of 2.00 ml, minimum compliance of 0.00 ml, and a unit gradient in ml. The results were considered normal when the output values were as follows: a compliance of 0.3 to 1.3 ml, a pressure of ±50 daPa, and a unit gradient of 1 to 2 ml. The results were classified according to Jerger’s curves as follows: type ‘A’ (normal compliance and pressure), type ‘B’ (without compliance peak and abnormal negative pressure), and type ‘C’ (normal compliance and abnormal negative pressure) [20]. The ASIR test was carried out using MT10 equipment (Interacoustics, Denmark). The ASIR was measured with trains of tone bursts between 70 and 110 dB HL. Frequencies of 500, 1000, 2000, and 4000 Hz were used. The ASIR response was biphasic, with an initial positive plateau followed by a longer negative one. For stimuli below 80 dB HL, the pattern of the reflex was monophasic, with a single positive peak. The presence of this peak was considered as a response, whereas its absence was classified as a lack of a response.

OAEs were elicited with ILO-V6 Otodynamics equipment (Otodynamic Limited, United Kingdom). The test was performed using a probe with two transducers and one microphone. Transient OAEs (TEOAEs) were produced by a brief click (80 µs) with a peak intensity of 80±5 dB SPL at frequencies of 1000, 2000, 3000, 4000, 6000 and 8000 Hz. The stability and sensitivity of this test is between 90 and 100%. In the distortion product OAEs (DPOAEs), the stimuli consisted of two pure tones (F1–F2) of frequencies ranging from 1000 to 8000 Hz, at an intensity of 70 dB SPL. The stimulus, signal to noise ratio, and emission reproducibility were recorded. A response was considered satisfactory when the signal was 5 dB above noise level with a maximum 60 s response time gate.

ABRs and ASSRs were studied in sleeping infants in a soundproof room using the AUDIX system (Neuronic Mexicana, S.A, México City). The ABR test was performed 100 µs monaural rarefaction clicks with intensities between 20 and 100 dB HL (hearing level), with a repetition rate of 11.2 Hz. An average of 2000 stimuli and a sweep time of 15 ms were used. The signal was recorded with an Ag/AgCl electrode disk in Cz with mastoids M1 and M2 as reference; impedance was kept under 5 kOhms in all electrodes. Two channels for ipsi- and contralateral registers were used. Amplifier gain was 100,000, with low-bandpass filters at 100 Hz and high-bandpass filters at 3000 Hz. The latency and amplitude of waves I to V and interpeak latencies I–III, III–V and I–V were obtained. A test was considered normal when the threshold for wave V was 30 dB HL; mild alterations were considered between 40 and 50 dB HL, moderate between 60 and 70 dB HL and severe when the threshold was higher than 80 dB HL.

The ASSR study was carried out with intensities between 20 and 100 dB HL in 5 to 10 dB steps. Stimuli were a combination of five carrier tones: 500, 1000, 2000, 4000 and 8000 Hz modulated in amplitude at rates: 95, 98, 101 and 105 Hz. Electrodes were placed in the mastoids (M1 or M2) as an active channel, and Cz was used as reference. Impedance was kept under 5 kOhms in all electrodes. Amplifier gain was 100,000 with low-cut filters at 10 Hz and high-cut filters at 300 Hz; the analysis period was 1.37 ms, and approximately 24 epochs were averaged. The frequency spectrum was obtained with the Fast Fourier Transform. Each spectral peak was considered as a vector in an X–Y coordinate system, where its length represented the amplitude of the spectral peak. The level of significance between the signal and noise spectral components was determined with Hotelling’s T2-test [21]. The significance level for the statistical detection of a signal was p<0.05. The ASSR thresholds were analyzed and classified as normal or abnormal by comparison with normal threshold values during the first year of life [22].

Hearing loss severity was evaluated based on calculated with the pure tone average (PTA) [14], [23], and was modified for pediatric age [24]. Hearing loss was classified as mild when the PTA threshold increased between 10 and 15 dB HL; moderate when PTA increased between >15 and 40 dB HL; severe when PTA increased between >40 and 60 dB HL, and profound when PTA increased >60 dB HL. When only mild abnormalities were found in one or two frequencies, hearing loss was classified as minimally altered.

Infants with profound hearing loss used a behind the ear (BTE) hearing aid with digital technology in the right ear; the left ear was not stimulated and was used as a control. The digital BTE hearing aid was adjusted with software (Aventa Standalone 2.2 version 2002–2006 GN Resound, U.S.) in 6 frequency bands (250, 500, 100, 2000, 4000 and 6000 Hz), and the maximum gain was established according to the threshold. To adjust the BTE hearing aids to each particular infant, an instant mold was made for the right ear. Indications for the use of BTE hearing aids were 4 to 6 hours a day in comfort program and volume set to 4 (maximum volume). Statistical analyses of the differences between auditory thresholds at the first and second evaluations of the two ears were carried out using Student’s t-test for paired samples.

## Results

Of the 378 infants evaluated, only 13 (3.4%) presented bilateral sensorineural severe to profound hearing loss. The study group consisted of 7 females and 6 males; 6 premature infants and 7 full-term infants. Premature infants had a gestational age of 35±1.6 weeks and full-term infants 39.2±1.2 weeks. At the first evaluation, the premature mean age was of 4.4±1.4 months, and the full-term mean age was 4.9±1.7 months. Tympanometry tests were carried out in all infants in the study group, and all results were normal (curve type A). The ASIR was absent in all frequencies evaluated.

### Otoacoustic Emissions

**Figure 1:**
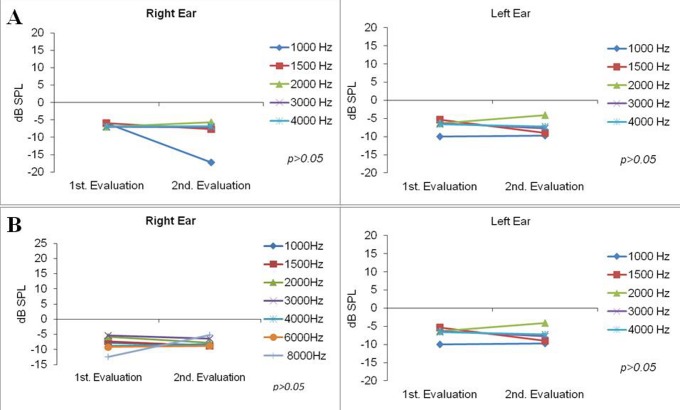
Otoacoustic emissions. (A) Transient otoacoustic emissions values were obtained in both ears at the following frequencies: 1000, 1500, 2000 and 4000 Hz in the first and second evaluations (after using a hearing aid in the right ear). (B) Distortion product otoacoustic emissions values were obtained in both ears at the following frequencies: 1000, 1500, 2000, 3000, 4000, 6000 and 8000 Hz in the first and second evaluations. No significant differences were found (p>0.05).

The TEOAE and DPOAE tests were carried out in all 13 infants. In the basal test, all infants exhibited no response in any of the frequencies. In the second evaluation, after six months of right ear HA use, OAEs showed no significant differences (p>0.05) (Figure 1).

### Auditory Brain Responses

#### a) Latencies

In the first evaluation, nine infants (69.2%) exhibited ABRs with mean latency in the right ear as follows: for wave I, 2.66±0.54 ms; wave II, 3.67±1.24 ms; wave III, 5.13±0.98 ms; wave IV, 6.87±0.89 ms; and wave V, 7.36±1.59 ms. Only 4 infants (30.8%) showed an absence of waves I to V when maximum stimulation (100 dB) was applied. In the second evaluation (after right ear HA use), all ABR waves were present in 9 infants (69.2%), and other infants (7.7%) presented waves IV and V; ABRs elicited no response in 3 infants (23.1%). The mean latencies in the right and left ears are shown in Table 1. No significant differences were observed between the right and left sides and between the first and second evaluations in either ear (p>0.05).

**Table 1:** Auditory Brain Responses (Latencies).

Ear	Evaluation	Waves					Inter-wave latencies		
		I	II	III	IV	V	I–III	III–V	I–V
Right	First	2.66 (0.54)	3.67 (1.23)	5.13 (0.98)	6.87 (0.89)	7.36 (0.76)	2.25 (0.47)	2.23 (0.63)	4.48 (0.52)
	Second	2.57 (0.34)	3.26 (0.38)	4.87 (0.73)	6.43 (0.61)	7.08 (0.61)	2.30 (0.65)	2.21 (0.71)	4.51 (0.52)
Left	First	2.43 (0.30)	3.15 (0.16)	4.71 (0.61)	6.35 (0.70)	7.15 (0.72)	2.28 (0.74)	2.43 (0.63)	4.71 (0.84)
	Second	2.43 (0.27)	3.02 (0.33)	4.40 (0.32)	6.11 (0.48)	6.80 (0.58)	1.97 (0.74)	2.43 (0.70)	4.40 (0.71)

Mean latencies in milliseconds (standard deviation).

Second evaluation after six months of right ear hearing aid use.

No significant difference between two evaluations and right and left side *p>0.05.*

#### b) Inter-wave latencies

In the first evaluation, only 9 infants (69.2%) presented waves I, III and V that allowed the assessment of inter-wave latencies. The mean values for the right and left ears are shown in Table 1. There were no significant differences between the first and second evaluations after right ear HA use in any of the control left ears (p>0.05).

#### c) Thresholds

In the first right ear evaluation, wave V was present in 9 infants (69.2%). To calculate the mean threshold, a numerical value of 110 dB was given to the infants with no response at 100 dB. The mean hearing threshold was 90.0±15.8 dB. In the second evaluation after right ear HA use, a significant decrease to 80.0±19.1 dB (p = 0.003) was observed. In the left ear, 7 infants (53.9%) presented wave V. The mean hearing threshold was 94.6±17.1 dB. In the second evaluation, the threshold decreased to 87.7±17.8 dB (p = 0.069). However, in the first evaluation of the right side, the ratio of wave V absence in preterm/term infants was one:three. On the left side, the no-response ratio observed was three:three. All infants without a response in the initial evaluation had a response in the second evaluation (Table 2).

**Table 2:** Auditory Brain Responses (Hearing Thresholds).

Subject	GA	First Evaluation		Second Evaluation	
		Right ear	Left ear	Right ear	Left ear
1	31	90	NR 100	70	100
2	36	NR 100	NR 100	80	100
3	36	90	90	80	90
4	40	70	70	70	80
5	31	90	80	80	80
6	42	80	100	80	80
7	39	NR 100	NR 100	NR 100	NR 100
8	38	80	90	70	90
9	38	90	70	70	60
10	41	NR 100	NR 100	NR 100	NR 100
11	33	70	70	50	60
12	32	70	NR 100	60	70
13	38	NR 100	NR 100	NR 100	NR 100
Mean (SD)	36.5(3.7)	90.0* (15.8)	94.6 (17.3)	80.0* (19.1)	87.6 (17.8)

GA = gestational age in weeks.

NR = No Response.

Hearing thresholds in decibels Hearing Level (dB HL).

Second evaluation after six months of hearing aid use (Right Ear).

Right ear, significant difference between two evaluations *p = 0.003.

Left ear; no significant differences between two evaluations *p = 0.07.*

### Auditory Steady State Responses

In the first evaluation, the mean hearing thresholds for the right ear in the frequencies tested were as follows : 500 Hz, 85.0±16.3 dB; 1000 Hz, 83.5±19.1 dB; 2000 Hz, 84.0±16.1 dB; 4000 Hz, 89.2±17.1 dB; and 8000 Hz, 90.4±15.9 dB. After right ear HA use, the mean thresholds of all frequencies decreased to 75.7±26.9 dB, 77.3±17.9 dB, 75.4±21.6 dB, 72.3±27.1 dB, and 79.6±19.6 dB, respectively. Only one significant decrease in the mean hearing threshold in the 4000 Hz frequency (p = 0.013) was found (Table 3). There were no significant differences between the first and second evaluation in any of the measured frequencies (p>0.05) in the left ear (Table 3).

**Table 3:** Auditory Steady State Responses (Hearing Thresholds).

	500 Hz				1000 Hz				2000 Hz				4000 Hz				8000 Hz			
	Firstevaluation		Secondevaluation		Firstevaluation		Secondevaluation		Firstevaluation		Secondevaluation		Firstevaluation		Secondevaluation		Firstevaluation		Secondevaluation	
Subject	RE	LE	RE	LE	RE	LE	RE	LE	RE	LE	RE	LE	RE	LE	RE	LE	RE	LE	RE	LE
1	80	100	100	70	100	100	100	90	90	100	80	70	100	100	50	60	100	100	80	100
2	100	100	100	100	70	100	70	50	80	50	100	50	100	100	100	100	100	100	100	100
3	100	100	100	100	90	90	90	90	100	80	100	80	100	50	100	50	100	90	100	90
4	100	100	80	70	100	80	80	90	70	100	70	90	100	90	100	100	60	100	80	100
5	80	100	40	100	60	100	80	85	100	60	50	70	90	90	100	100	100	80	60	100
6	50	90	30	70	90	70	70	30	80	80	90	50	80	50	30	100	60	60	100	100
7	NR	NR	100	100	NR	NR	90	NR	NR	NR	100	100	NR	NR	NR	NR	NR	NR	80	NR
8	75	70	75	70	75	100	75	100	50	80	50	80	60	60	60	60	75	70	75	70
9	70	60	70	40	40	40	80	50	70	70	80	60	50	80	40	50	100	80	100	60
10	NR	NR	100	100	NR	NR	100	90	80	80	100	80	NR	NR	90	90	100	NR	100	NR
11	80	30	30	35	90	80	30	30	70	70	30	40	80	70	30	30	80	70	30	30
12	70	60	80	90	70	60	70	80	100	40	80	100	100	50	80	90	100	60	80	90
13	NR	NR	90	100	NR	NR	70	NR	NR	NR	70	100	NR	NR	80	NR	NR	NR	70	NR
Mean(SD)	85(16.3)	85.4(23)	76.5(26.9)	80.4(23.3)	83.5(19.1)	86.2(19.4)	77.3(17.9)	75.8(26.1)	83.8(16.1)	77.7(19.6)	76.9(22.5)	74.6(20.3)	89.2*(17.1)	80(21.2)	73.8*(28.1)	79.2(25.3)	90.4(15.9)	85.4(16.1)	81.2(20.4)	87.7(21.7)

Hz = Hertz.

RE = Right Ear.

LE = Left Ear.

NR = No Response.

Significant difference between two evaluations *p = 0.013.

## Discussion

The aim of this study was to analyze the effects of early HA use (before six months of age) on auditory function in a group of infants. Main finding of our study is that in infants with PBI and severe to profound hearing loss, the use of a HA decreased the hearing threshold evaluated based on ABR and ASSR tests, with no modifications in auditory receptor function, as assessed based on OAEs.

All the infants in our study presented with severe to profound sensorineural hearing loss without OAEs in the basal evaluation. The causes of hearing loss in our group of infants with PBI were similar to those found in other studies, and included hyperbilirrubinemia, ototoxic antibiotics, and hypoxic-ischemic encephalopathy [25]–[27]. All these factors affect the cochlear OHCs with mild alterations to inner hair cells (IHCs) [28]. It is known that the absence of OAEs is indicative of OHC damage with mild and moderate hearing loss (30 to 60 dB), but does not indicate hearing threshold alterations. Therefore, it is possible that infants in our study with severe to profound hearing loss presented both OHC and IHC dysfunction, but with enough IHC functionality to maintain residual hearing. This is important because OHC damage characteristically produces increases in hearing threshold and decreases in frequency selectivity [6].

We propose that the preserved function of those IHCs may be sufficient for environmental sounds (with the amplification of the HA) to stimulate the receptor, auditory pathways and auditory cortex. This may also explain why infants with severe to profound hearing loss with total damage to the OHCs and partial damage to the IHCs presented no OAEs, and yet IHC and auditory pathway activation were enough to produce action potentials that allowed ABR and ASSR evaluations. In agreement with this concept, Kempt proposes two types of sensory hearing loss, transmissive and transduction types, depending on the alteration of the OHCs or IHCs, respectively [28].

Auditory pathway properties were evaluated based on ABRs. These responses were present in nine of 13 infants in the initial evaluation; after six months of right ear HA use, the number of infants with ABR responses was the same. However, in the first evaluation of the right side, the proportion of wave V absence in preterm/term infants was one/three, while it was three/three on the left side. Independent of side or prematurity, all infants without a response in the initial evaluation had a response in the second evaluation. This can be explained as an effect of age, which allows for maturation and better myelination of the auditory pathways with the appearance of ABR in the second evaluation.

When the hearing thresholds were compared, the right side decreased 10 dB with no significant changes in the latencies or amplitudes of Waves I to V. In the left ear, no significant decrease in the hearing threshold was observed between the two evaluations. Therefore, we postulated that the effect observed on the right side was due to the six months of HA use. It is possible that hearing pathway maturation has some contribution to this effect, as was observed in the analysis of the proportion of ABR absence.

Although many studies have been carried out to analyze plastic reorganization of auditory cortex after experimental and human hearing loss and subsequent correction with cochlear implants; only a few studies have analyzed the plastic changes of auditory pathways after HA [29]–[31].

The decrease in hearing threshold observed with ABRs was also found for ASSRs, where the hearing threshold in the 4000 Hz frequency decreased from 89 dB to 72 dB after right ear HA use. This finding is relevant because it is known that the hearing threshold obtained with ABRs only evaluates a narrow frequency spectrum, between 2000 and 4000 Hz. In addition, it has been reported that ABR generators of Wave V are located in the mesencephalic region at the level of the inferior colliculus. Previously published, inconclusive results indicated that ASSR electrical sources might be localized in the brain stem and cortex at the level of left and right supratemporal plane [32].

The decrease in hearing threshold found in both tests but only on the right side after HA use suggests an increase in ABR and ASSR amplitude to the same intensity of the stimulus; the increase in amplitude may be due to an increase in the number of auditory pathway fibers synchronically activated or to an increase in synaptic efficacy in some nuclei of this pathway [18], [30].

Experimental models of hearing loss have shown decreases in the number or size of neurons in cochlear nucleus and trapezoid body [33], [34]. Some changes in neuronal morphology have been reported in the superior olivary complex and lateral lemniscus [35]. In other auditory pathway structures, bilateral deafness is required to induce synaptic density changes in the inferior colliculus [36]. Conversely, functional changes have been observed even in unilateral deafness. These changes are relevant to explain the decrease in threshold in our group of infants. In normal hearing animals, cochlear stimulation activates nearly 30% of neurons in the inferior colliculus; this percentage increases to 70% in unilateral deaf adult animals, and 90%, when the cochlear lesion is produced during the neonatal period [37]. In addition, a reduction in temporal neuronal resolution has been observed in neonatal bilateral deafened animals in relation to normal or unilateral deaf animals used as controls.

On the other hand, the maturation pattern of electrically evoked auditory brainstem responses in deaf infants during the first two years after cochlear implant has been compared with the ABR maturation pattern of normal hearing infants [30]. These authors reported a decrease in wave V latency and auditory threshold only when the cochlear implant was adapted to infants with early-onset deafness; infants with late-onset deafness showed no modifications in latencies. They concluded that the modifications in latency and wave V threshold are due to plastic changes in auditory pathways that may include increased myelination or synaptic efficiency. These plastic changes may be similar to those found in our study, where a decrease in wave V threshold in the ABR assessment was present after right ear HA use.

The present study included a small sample size. As a result, the conclusions should be considered with caution. Additionally, compliance with HA use could only be measured indirectly with mothers’ self-reports of the duration of HA use.

In conclusion, auditory function in infants is modified after early stimulation with HA before 6 months of age, causing a decrease in the hearing threshold determined based on ABR and ASSR evaluations, with no modifications in auditory receptor function as evaluated based on OAEs.
